# Seroprevalence of Epstein-Barr Virus Infection in U.S. Children Ages 6-19, 2003-2010

**DOI:** 10.1371/journal.pone.0064921

**Published:** 2013-05-22

**Authors:** Jennifer Beam Dowd, Tia Palermo, Jennifer Brite, Thomas W. McDade, Allison Aiello

**Affiliations:** 1 Epidemiology and Biostatistics, CUNY School of Public Health at Hunter College, New York, New York, United States of America; 2 CUNY Institute for Demographic Research (CIDR), New York, New York, United States of America; 3 Graduate Program in Public Health, Department of Preventive Medicine, Stony Brook University, Stony Brook, New York, United States of America; 4 Public Health, CUNY Graduate Center, New York, New York, United States of America; 5 Cells to Society (C2S): The Center on Social Disparities and Health, Institute for Policy Research, Northwestern University, Evanston, Illinois, United States of America; 6 Department of Anthropology, Northwestern University, Evanston, Illinois, United States of America; 7 Department of Epidemiology, School of Public Health, University of Michigan, Ann Arbor, Michigan, United States of America; University of Hong Kong, Hong Kong

## Abstract

**Background:**

Epstein-Barr virus (EBV) is a common herpesvirus linked to infectious mononucleosis and multiple cancers. There are no national estimates of EBV seroprevalence in the United States. Our objective was to estimate the overall prevalence and sociodemographic predictors of EBV among U.S. children and adolescents aged 6–19.

**Methods:**

We calculated prevalence estimates and prevalence ratios for EBV seroprevalence using data from the 2003–2010 U.S. National Health and Nutrition Examination Survey (NHANES) for children aged 6–19 (n = 8417). Poisson regression was used to calculate multivariable-adjusted prevalence ratios across subgroup categories (sex, race/ethnicity, parental education, household income, household size, foreign-born, BMI, and household smoking).

**Findings:**

Overall EBV seroprevalence was 66.5% (95% CI 64.3%–68.7%.). Seroprevalence increased with age, ranging from 54.1% (95% CI 50.2%–57.9%) for 6–8 year olds to 82.9% (95% CI 80.0%–85.9%) for 18–19 year olds. Females had slightly higher seroprevalence (68.9%, 95% CI 66.3%–71.6%) compared to males (64.2%, 95% CI 61.7%–66.8%). Seroprevalence was substantially higher for Mexican-Americans (85.4%, 95% CI 83.1%–87.8%) and Non-Hispanic Blacks (83.1%, 95% CI 81.1%–85.1%) than Non-Hispanic Whites (56.9%, 95% CI 54.1%–59.8%). Large differences were also seen by family income, with children in the lowest income quartile having 81.0% (95% CI 77.6%–84.5%) seroprevalence compared to 53.9% (95% CI 50.5%–57.3%) in the highest income quartile, with similar results for parental education level. These results were not explained by household size, BMI, or parental smoking. Among those who were seropositive, EBV antibody titers were significantly higher for females, Non-Hispanic Blacks and Mexican-Americans, with no association found for socioeconomic factors.

**Conclusions:**

In the first nationally representative U.S. estimates, we found substantial socioeconomic and race/ethnic differences in the seroprevalence of EBV across all ages for U.S. children and adolescents. These estimates can help researchers and clinicians identify groups most at risk, inform research on EBV-cancer etiology, and motivate potential vaccine development.

## Introduction

Epstein-Barr virus (EBV), a member of the herpesvirus family, is one of the most common human viruses and once contracted persists for the lifetime of the person. EBV is generally transferred through saliva and can infect infants as soon as maternal antibody protection subsides. Primary infection can occur throughout the life course, with approximately 90% of the human population estimated to be infected [1]. However, the age of onset is thought to vary widely, with developed countries having higher ages at primary infection, most likely due to better hygienic conditions and other socioeconomic and demographic factors including household size and population density [2]. If primary infection occurs during early childhood, the virus generally causes no symptoms or is indistinguishable from other common, but mild, illnesses [2]. However, up to 50% of those who experience primary infection in later childhood or adolescence may contract infectious mononucleosis [3], and age at onset may be associated with EBV-related malignancies [2], [4]. Epstein-Barr was the first virus to be linked to cancer, and has been linked to nearly all cases of nasopharyngeal carcinoma and important subsets of Burkitt lymphomas, other non-Hodgkins lymphoma and gastric cancer [5], [6], [7], [8]. EBV infection or reactivation has also been associated with lupus [9], multiple sclerosis [10], and cardiovascular disease[11].

Despite its potential medical and public health burden, to our knowledge, no national population-based estimates of EBV prevalence in the U.S. exist. Previous studies focused on special populations, such as pregnant women [12], or were derived from relatively small sample sizes in limited geographic areas [13], [14], [15] that are therefore not generalizable to the country as a whole; some still-cited studies are more than forty years old [13], [16]. Reliable estimates of EBV prevalence are required to identify groups most at risk, inform research on EBV-cancer etiology, and motivate potential vaccine development. Given the previous findings of socioeconomic and racial/ethnic differences in similar infections[17], it is important to examine the prevalence of EBV across diverse socioeconomic and racial groups.

We analyzed publicly-available data from the National Health and Nutrition Examination Survey (NHANES) for the years 2003–2010 to estimate EBV seroprevelance for ages 6–19 in the U.S. and its association with sociodemographic variables, such as age, sex, race/ethnicity, and household education and income. We also examined the association of risk factors such as household size, smoking, and body mass index. To our knowledge, this is the first study to estimate EBV seroprevalence among U.S. children and adolescents with nationally representative data.

## Materials and Methods

### Ethics Statement

The current study was secondary analysis of de-identified public data was determined to be exempt by the Institutional Review Board of Hunter College.

### Data

Data come from the 2003–2010 U.S. National Health and Nutrition Examination Survey (NHANES), a nationally representative, cross-sectional survey of the non-institutionalized U.S. population with oversamples of the elderly, non-Hispanic blacks, and Mexican Americans. NHANES is conducted annually and data are publicly released in two-year waves (2003–2004, 2005–2006, 2007–2008, 2009–2010), providing interview, examination, and laboratory measures. Trained interviewers, using a computer-assisted personal interview system, interviewed participants at home. Participants were subsequently asked to attend a mobile examination center, where they were asked to complete additional questionnaires, undergo various examinations, and to provide biological specimens, including blood and urine. For children under 15 years of age, a proxy interview with a parent was conducted. Additional details of the NHANES survey design have been published elsewhere [18].

### Measures

#### Serological Testing

EBV antibody testing was conducted among children 6–19 years who participated in NHANES between 2003–2010 and had stored serum samples available (n = 9302). EBV VCA IgG antibody was measured using a commercial enzyme immunoassay kit (Diamedix, Miami, FL). Data were recorded as Positive (EIA≥1.10), Negative (EIA index≤.90), or Equivocal (0.90<EIA≥1.09). The sensitivity of the assay was 96.6% and the specificity was 97.7%. All QA/QC procedures recommended by the manufacturer were followed. Documentation can be accessed at (http://www.cdc.gov/nchs/nhanes/nhanes2009-2010/SSEBV_F.htm). Equicovals (n = 46) were excluded from analysis. Six respondents did not have sufficient serum quantity for the assay, and 833 were excluded for missing values on covariates, leaving a final sample of 8417.

### Covariates

Several sociodemographic variables were examined as predictors of EBV. Reported family income was adjusted for inflation to the year 2000 using the Consumer Price Index then divided into income quartiles for analysis. Race/ethnicity was self-reported and classified as non-Hispanic white, non-Hispanic black, Mexican-American, and other race/ethnicity (non-Hispanic white as the reference category). Education was measured as the highest level of education achieved by the head of household and coded as less than high school, high school completion, or greater than high school completion. Household size was coded as <5, 5–6, or >6. Additional factors that might impact immune function and susceptibility to EBV were also considered. Since children who are immigrants may have encountered a different pathogen environment in utero and early life, we included a variable indicating whether the child was born inside or outside of the U.S. Exposure to environmental tobacco smoke may be deleterious to a child’s immune system, therefore we included a variable indicating whether the parent reported at least one household smoker. An increase in adipose tissue has been shown to alter certain immune parameters [19], [20]. BMI was calculated as (kg/m^2)^ from measured height and weight during the exam and converted to age and sex specific z-scores based on the 2000 CDC growth charts.[21]. BMI was then coded as normal/under, overweight, and obese.

### Statistical Analysis

Mean prevalence across age groups (6–8, 9–11, 12–14, 15–17, 18–19) was calculated and plotted by race/ethnicity and income quartiles. Next, unadjusted prevalence ratios were calculated directly from tabulation of prevalence rates across categories of covariates. Multivariable Poisson regression was used to determine whether the key sociodemographic variables (age, sex, and race/ethnicity, household income, education, and household size) as well as physical risk factors (BMI and household smoking) were predictors of EBV seroprevalence in fully adjusted models. Results are reported in prevalence estimates and prevalence ratios. As a secondary analysis, we also examined the association of our covariates with continuous (logged) EBV antibody titers among those seropositive using linear regression. All analyses were adjusted for sample weights and NHANES complex survey design using Stata (11.2).

## Results

**Figure 1:**
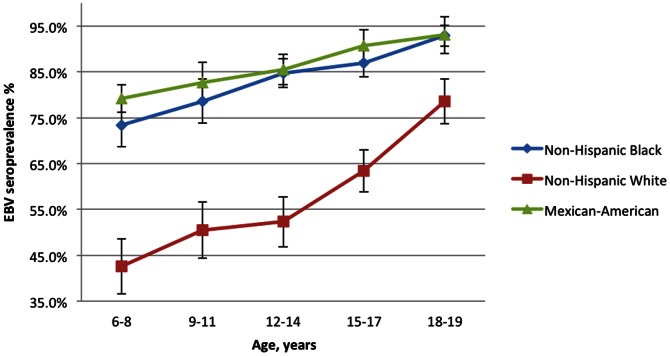
Mean EBV Seroprevalence by age and race/ethnicity, National Health and Nutrition Examination Survey, 2003–2010. Bars indicate 95% confidence intervals.

**Figure 2:**
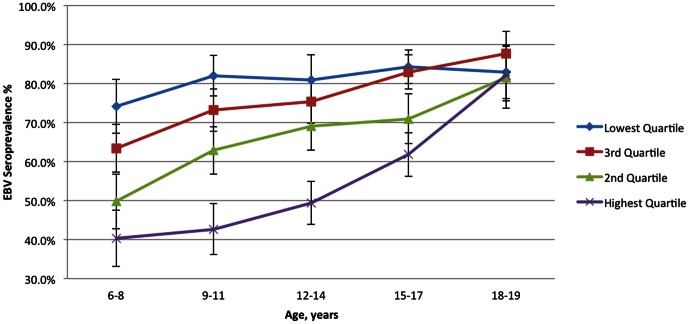
Mean EBV Seroprevalence by age and income quartile, National Health and Nutrition Examination Survey, 2003–2010. Bars indicate 95% confidence intervals.

Table 1 shows descriptive characteristics of the overall sample. The overall seroprevalence of Epstein-Barr virus (EBV) for children aged 6–19 in the United States was 66.5% (95% CI 64.3%–68.7% (Table 2)). This ranged from 54.1% among 6–8 year-olds (95% CI 50.2%–57.9%) to 82.9% (80.0%–85.9%) among 18-19-year-olds. Males were slightly less likely to be infected than females (64.2% vs. 68.9%). Higher unadjusted prevalence was seen for non-white children and those with lower household income and education, children born outside the U.S., obese children, children with a smoker in the house, and children living in a larger household. Figures 1 and 2 illustrate trends in seroprevalence across age broken down by race/ethnicity (Figure 1) and income quartile (Figure 2).

**Table 1:** Weighted descriptive characteristics, ages 6–19 NHANES 2003–2010 (n = 8417).

	Mean/ proportion	95% C.I
Seropositive for EBV	66.5%	(64.3–68.7)
Age	12.80	(12.6–12.9)
Male	51.8%	(50.3–53.2)
Female	48.2%	(46.8–49.7)
Race/ethnicity		
White	60.0%	(55.8–64.1)
Black	14.6%	(12.3–17.0)
Mexican American	13.6%	(10.8–16.3)
Other race	11.8%	(9.8–13.8)
Weight status		
Under/normal	58.8%	(57.1–60.5)
Overweight	19.7%	(18.5–20.8)
Obese	14.2%	(13.0–15.4)
Household income		
4th (Lowest) quartile	17.8%	(15.8–19.9)
3rd quartile	22.9%	(21.0–24.7)
2nd quartile	23.2%	(21.2–25.3)
1st (Highest) quartile	36.0%	(32.8–39.3)
Household reference education		
<High school	19.8%	(17.8–21.7)
Complete high school	25.0%	(22.8–27.3)
>High school	55.2%	(52.7–57.7)
Country of birth		
US	93.3%	(92.3–94.2)
Other country	6.7%	(5.8–7.7)
Household smoker		
No smoker in household	80.7%	(78.5–83.0)
Smoker in household	19.3%	(17.0–21.5)
Household size		
<5	80.7%	(78.5–83.0)
5–6	34.2%	(32.6–35.8)
>6	8.5%	(7.1–9.9)

**Table 2:** Epstein-Barr seroprevalence among US children, ages 6–19, 2003–2010.

Characteristic	Prevalence estimate	95% CI	Prevalence ratio	(95% CI)
Total	66.5%	(64.3–68.7)		
**Age, years**				
6–8	54.08%	(50.2–57.9)		
9–11	60.75%	(56.9–64.6)		
12–14	64.15%	(60.4–67.9)		
15–17	71.70%	(68.6–74.8)		
18–19	82.90%	(80.0–85.9)		
**Sex**				
Female	68.9%	(66.3–71.6)	1.00	
Male	64.2%	(61.7–66.8)	0.93	(0.89–0.97)
**Race/ethnicity**				
White	56.9%	(54.1–59.8)	1.00	
Black	83.1%	(81.1–85.1)	1.46	(1.38–1.55)
Mexican American	85.4%	(83.1–87.8)	1.50	(1.42–1.59)
Other race	72.9%	(68.7–77.0)	1.28	(1.19–1.38)
**Household income**				
4th (Lowest) quartile	81.0%	(77.6–84.5)	1.50	(1.40–1.61)
3rd	75.7%	(72.3–79.2)	1.40	(1.30–1.51)
2nd	65.7%	(62.0–69.4)	1.22	(1.12–1.33)
1st (Highest) quartile	53.9%	(50.5–57.3)	1.00	
**Household education**				
<High school	83.5%	(80.5–86.5)	1.41	(1.34–1.49)
Complete high school	69.5%	(66.2–72.8)	1.18	(1.12–1.24)
>High school	59.0%	(56.5–61.5)	1.00	
**Country of Birth**				
US	65.2%	(62.9–67.4)	1.00	
Other country	84.5%	(79.8–89.2)	1.30	(1.22–1.37)
**Weight status**				
Under/normal	63.5%	(60.8–66.3)	1.00	
Overweight	64.7%	(61.1–68.2)	0.98	(0.92–1.04)
Obese	71.7%	(66.5–76.8)	1.09	(1.01–1.17)
**Smoking in household**				
No smoker in household	64.4%	(62.1–66.7)	1.00	
Smoker in household	75.4%	(71.6–79.2)	1.17	(1.11–1.24)
**Household size**				
<5	64.1%	(61.7–66.5)	1.00	
5–6	67.6%	(64.7–70.5)	1.06	(1.02–1.11)
>6	82.7%	(78.9–86.5)	1.30	(1.24–1.37)

Models in Table 3 were adjusted first for age, sex, and race (Model 1) and then the full set of covariates (Model 2). In the fully adjusted model, socioeconomic status and other risk factors accounted for a portion but not all of the increased EBV seroprevalence for non-white children and adolescents [PR for blacks: 1.33 (95% CI: 1.26–1.41); PR for Mexican Americans: 1.37 (95% CI: 1.28–1.46); PR for “other” races: 1.22 (95% CI: 1.13–1.31)]. Parental socioeconomic status remained an important predictor of EBV net of other factors: children with parents who completed less than high school had higher levels of EBV seroprevalence compared to those with parents who had completed more than high school [PR: 1.10 (95% CI: 1.03–1.17)] (Table 3), and the lowest quartile of household income was associated with a striking 23% higher prevalence compared to those in the highest income quartile [PR 1.23, CI: 1.15–1.31] (Tables 2 and 3). While being obese compared to normal weight was still associated with a 3% higher EBV prevalence, the association was no longer statistically significant in the adjusted models (PR 1.03, 95% CI 0.96–1.11). Larger family size and having a smoker in the household remained significantly associated with higher seroprevalence in fully-adjusted models, suggesting that family size and smoking may have an independent impact on acquiring EBV that is not accounted for by other sociodemographic factors.

**Table 3:** Adjusted prevalence ratios of EBV seroprevalence in children ages 6–19, NHANES 2003–2010 (n = 8417).

	(1)	(2)
	Adjusted prevalence ratio (95% CI)	Adjusted prevalence ratio (95% CI)
Age	1.04***	1.04***
	(1.03 – 1.04)	(1.03 – 1.04)
Male	0.94**	0.94**
	(0.90 – 0.98)	(0.90 – 0.99)
Race/ethnicity		
White	1.00	1.00
Black	1.46***	1.33***
	(1.38 – 1.55)	(1.26 – 1.41)
Mexican American	1.53***	1.37***
	(1.45 – 1.63)	(1.28 – 1.46)
Other race	1.30***	1.22***
	(1.21 – 1.40)	(1.13 – 1.31)
Foreign born		1.07*
		(1.01 – 1.14)
Weight status		
Normal/Underweight		1.00
Overweight		0.98
		(0.93 – 1.04)
Obese		1.03
		(0.97 – 1.11)
Income quartiles		
1st (highest)		1.00
2nd		1.13**
		(1.04 – 1.23)
3rd		1.21***
		(1.12 – 1.31)
4th (lowest)		1.23***
		(1.15 – 1.31)
Household reference's education		
> high school		1.00
completed high school		1.07*
		(1.02 – 1.13)
> high school		1.10**
		(1.03 – 1.17)
Smoker in household		1.13***
		(1.08 – 1.19)
Household size (ref = <5)		
5–6		1.06*
		(1.01 – 1.10)
>6		1.14***
		(1.07 – 1.22)

*** p<0.001, ** p<0.01, * p<0.05

Higher EBV antibody titers have been associated with increased risk of onset of EBV-related malignancies[22], [23], therefore we also examined the correlates of (logged) EBV antibody titers among those who were seropositive as a secondary analysis. In fully adjusted models, males had significantly lower EBV antibody titers compared to women, and Blacks and Mexican-Americans had higher antibody titers compared to whites. No differences in antibody titer were seen by age, BMI, parental education or household income.

## Discussion

In the United States, Epstein-Barr virus seroprevalence was estimated to be approximately 66.5% among children ages 6–19 (58.5% for children 6–12 and 73.4% for those 12–19) in the United Sates for 2003–2010. National estimates of EBV seroprevalence for any country are rare, and to our knowledge these are the first nationally representative EBV prevalence estimates for the United States. McDade, et al. found an EBV prevalence of 80.1% in a group of 9–13 year olds participating in the Great Smoky Mountains Study [15], while Matro, et al, found a 67% seroprevalence among a sample of children aged 3–17 years seen in Georgia hospitals for illness or routine care[14]. This seroprevalence is lower than that found in developing countries worldwide, where it is estimated more than 90% of the population is infected in early childhood [24], [25], [26], [27]. The seroprevalence is comparable to those found in non-representative samples in Western, developed countries—in England, 45% of 5–9 year olds [28] and Germany, 74% for 3–17 year olds[14]. Given the scarcity of national estimates, our findings will provide a valuable baseline for tracking trends in future EBV prevalence among U.S. children.

We observed large differences in EBV seroprevalance by race/ethnicity, with Mexican American children having the highest seroprevalence (85.4%) followed by non-Hispanic blacks (83.1%), and whites (56.9%). The observed differences by race/ethnicity were slightly reduced but not explained by household socioeconomic status, with both remaining strong independent predictors of EBV risk. These differences were also not accounted for by risk factors such as household size, BMI, or having a smoker in the household. EBV seroprevelance differences by race/ethnicity have previously been identified in several studies from the 1970s, including between black and white U.S. military cadets [29] and among different ethnic groups in Hawaii [13]. The significant differences in EBV seroprevalence by household income and education as well as race/ethnicity among U.S. children are consistent with differences previously identified for cytomegalovirus (CMV), herpes simplex virus -1 (HSV-1), *Helicobacter Pylori,* Hepatitis A, and Hepatitis B [17], [30]. It is well known that socioeconomic status (SES) is consistently associated with adult health outcomes. The timing of primary infection with EBV may be important for the etiology of EBV-related malignancies [31], and thus differences in the timing of acquisition by social variables could be important for understanding later links to cancer onset in adulthood. Specifically, EBV-related Hodgkins lymphoma risk in young adulthood is associated with infectious mononucleosis indicative of delayed acquisition of EBV [32], while early-life acquisition is believed to be associated with Burkitt’s lymphoma and nasopharyngeal carcinoma [22], [33], [34]. The observed differences in EBV seroprevalence and antibody response by race/ethnicity identified in this study may help shed light on different prevalence and age patterns of EBV-related diseases by race/ethnicity in the U.S [35].

Future work should examine the sources of differential rates of seropositivity among U.S. children. With current NHANES data, it is impossible to distinguish whether different rates are a result of increased *exposure*, increased *susceptibility,* or both. EBV is believed to be primarily transmitted through saliva, though may also be transmitted via blood transfusions, sexual intercourse, or urine [31]. While household size was associated with an increased likelihood of infection, it did not alter the relationship between SES or race/ethnicity and EBV seroprevalence. It is possible that in groups with historically higher rates of infection who predominantly live and work together, higher levels would persist over time. Environmental factors associated with socioeconomic status, such as household crowding or use of public transportation, could contribute directly to exposure risk. Suppressed immune function as a result of stress, poor nutrition, smoking, or other environmental exposures could increase susceptibility to infections given equal levels of exposure. Low social status as well as indicators of psychosocial stress can impact risk of respiratory infections in humans and other primates in experimental studies [36], [37], [38], [39], [40]. Less is known about the links between social status, stress, and susceptibility to infections in the broader U.S. population. Low social class was associated with lower secretory immunoglobulin (sIgA), cited as a first line of defense against infection, in a large community sample in Scotland [41]. Taken together, these studies suggest that psychological stress associated with lower social status could down-regulate various aspects of the cellular immune response, increasing susceptibility to infection. Future work should aim to build evidence regarding the sources of such early differences in infection rates.

EBV is the cause of infectious mononucleosis and has been linked to certain types of cancers. The burden of EBV in the US population ages 6–19 is substantial. Large racial/ethnic disparities in EBV are not explained by socioeconomic status or other factors that could impact transmission such as household size. Future work should examine the factors associated with race/ethnic and socioeconomic differences in EBV acquisition prevalence among children in the US.
